# Applying the D50 disease progression model to gray and white matter pathology in amyotrophic lateral sclerosis

**DOI:** 10.1016/j.nicl.2019.102094

**Published:** 2019-11-28

**Authors:** Robert Steinbach, Meerim Batyrbekova, Nayana Gaur, Annika Voss, Beatrice Stubendorff, Thomas E. Mayer, Christian Gaser, Otto W. Witte, Tino Prell, Julian Grosskreutz

**Affiliations:** a Hans Berger Department of Neurology, Jena University Hospital, Jena, Germany; b Center for Healthy Ageing, Jena University Hospital, Jena, Germany; c Department of Neuroradiology, Jena University Hospital, Jena, Germany

**Keywords:** Amyotrophic lateral sclerosis, Magnetic resonance imaging, Voxel-Based Morphometry, D50 model, Disease progression, ALS, Amyotrophic Lateral Sclerosis, ALSFRS-R, ALS Functional Rating Scale (Revised), FWE, Family-Wise Error, GM, Gray Matter, MRI, Magnetic Resonance Imaging, PR, Progression Rate, TFCE, Threshold-Free Cluster Enhancement, VBM, Voxel-Based Morphometry, WM, White Matter

## Abstract

Therapeutic management and research in Amyotrophic Laterals Sclerosis (ALS) have been limited by the substantial heterogeneity in progression and anatomical spread that are endemic of the disease. Neuroimaging biomarkers represent powerful additions to the current monitoring repertoire but have yielded inconsistent associations with clinical scores like the ALS functional rating scale. The D50 disease progression model was developed to address limitations with clinical indices and the difficulty obtaining longitudinal data in ALS. It yields overall disease aggressiveness as time taken to reach halved functionality (D50); individual disease covered in distinct phases; and calculated functional state and calculated functional loss as acute descriptors of local disease activity. It greatly reduces the noise of the ALS functional rating scale and allows the comparison of highly heterogeneous disease and progression subtypes. In this study, we performed Voxel-Based Morphometry for 85 patients with ALS (60.1 ± 11.5 years, 36 female) and 62 healthy controls. Group-wise comparisons were performed separately for gray matter and white matter using ANCOVA testing with threshold-free cluster enhancement. ALS-related widespread gray and white matter density decreases were observed in the bilateral frontal and temporal lobes (*p* < 0.001, family-wise error corrected). We observed a progressive spread of structural alterations along the D50-derived phases, that were primarily located in frontal, temporal and occipital gray matter areas, as well as in supratentorial neuronal projections (*p* < 0.001 family-wise error corrected). ALS patients with higher overall disease aggressiveness (D50 < 30 months) showed a distinct pattern of supratentorial white matter density decreases relative to patients with lower aggressiveness; no significant differences were observed for gray matter density (*p* < 0.001 family-wise error corrected).

The application of the D50 disease progression model separates measures of disease aggressiveness from disease accumulation. It revealed a strong correlation between disease phases and in-vivo measures of cerebral structural integrity. This study underscores the proposed corticofugal spread of cerebral pathology in ALS. We recommend application of the D50 model in studies linking clinical data with neuroimaging correlates.

## Highlights

• The D50 disease progression model well characterized a cross-sectional ALS cohort.
• VBM reveled ALS-related widespread gray and white matter density decreases.
• A spread of structural alterations occurs along with D50 model derived disease phases.
• White-matter alterations were associated with higher disease aggressiveness.

## 1 Introduction

Amyotrophic lateral sclerosis (ALS) is a progressive multi-systemic neurodegenerative disorder, with most patients typically succumbing to respiratory failure 2–5 years from symptom onset (Dorst et al., 2018; Kiernan et al., 2011). Both ALS research and care are severely constrained by substantial intra- and inter-individual phenotypic heterogeneity and the lack of well-characterized and large longitudinal data sets. Biomarkers that can detect and reflect individual disease aggressiveness are therefore urgently needed. The potential of neuroimaging as a powerful and non-invasive biomarker for longitudinally monitoring neurodegenerative processes has been accepted within both the ALS and the wider neurodegenerative communities. Voxel-Based Morphometry (VBM) in particular has already demonstrated group-wise regional differences in cerebral tissue compartments of T1 images (Ashburner and Friston, 2000). In ALS, using VBM has revealed widespread loss of structural integrity (Bede and Hardiman, 2014; Chio et al., 2014; Pradat and El Mendili, 2014). However, results between studies and centers are often disparate; these inconsistencies have been ascribed to, among other things, methodological and recruitment differences and the substantial heterogeneity between ALS cohorts (Chen et al., 2018a; Shen et al., 2016). Several VBM studies have failed to show consistent correlations with disease progression using the revised ALS Functional Rating Scale (ALSFRS-R)-derived Progression Rate (PR). Clinical parameters that fully capture heterogeneity and reflect functional loss at both the individual and population level are needed to be able to fully utilize any biomarkers, neuroimaging or otherwise. While longitudinal data sets would be best-suited to performing such correlation analyses and monitoring these changes over time, such studies are often limited by the high dropout rates and poor longevity of patients suffering from ALS. Large-scale cross-sectional datasets, as for instance available from multi-center collaborations like the NiSALS consortium, may alternatively be used as “pseudo-longitudinal” substitutes (Steinbach et al., 2018; Turner et al., 2011).

The D50 model was recently developed to address limitations with existing clinical indices and to help describe and characterize both disease aggressiveness and phases of individual disease covered in a manner that is independent of the absolute time since onset at which a patient was assessed (Poesen et al., 2017; Prell et al., 2019). As such, the model 1) addresses phenotypic complexity, 2) helps reduce noise associated with the ALSFRS-R and 3) provides distinct descriptors of patients’ disease phase and aggressiveness.

We hypothesize that D50 model-derived parameters may provide a new and more insightful framework within which to interpret in vivo measures of cerebral structural integrity. Therefore, we performed VBM for both GM and WM segments of a well-characterized cross-sectional ALS cohort; results were analyzed in relation to clinical indices that were calculated using the D50 model.

## 2 Material and methods

### 2.1 Subjects

Written informed consent was obtained from individual participants prior to study commencement. This study was approved by the local Ethics committee (Nr 3633-11/12) and all experimental procedures were performed in accordance with the ethical standards defined in the 1964 Declaration of Helsinki and its later amendments. All participants were consecutively recruited from the Department of Neurology, Jena University Hospital. Eighty-five subjects with either definite, probable, laboratory-supported probable or possible ALS (Brooks et al., 2000) and 62 sex-matched controls (age 54 ± 12.8 years, range 21 - 77.6; 35 female) were enrolled. All ALS patients were examined and diagnosed by a trained neurologist prior to enrollment and during follow-up visits; patients were excluded if they displayed any clinically relevant symptoms of dementia. ALS patients with any comorbidities that could affect motor performance or cognition were also excluded, as were patients with manifestations of either juvenile ALS or primary lateral sclerosis. Detailed clinical data are presented in Table 1.

**Table 1:** Demographic and Clinical Data for Patients with ALS (*n* = 85).

demographic/clinical parameter	

Age [in years, mean ± SD, range]	60± 11.5 (27.3 - 78.8)
Gender [male/female, n, %]	49/36, 57.6%/42.4%
Riluzole [yes/no, n, %]	65/20, 76.4%/23.5%
Disease duration [in months, mean ± SD, range]	20 ± 21.6 (4 −136)
Onset-type [limb/bulbar, n, %]	61/24, 71.8%/28,2%
ALSFRS-R total score [mean ± SD, range]	38.1 ± 6.9 (15 - 47)
PR [points lost per month, mean ± SD, range]	0.69 ± 0.55 (0.06 – 2.71)
rD50 [mean ± SD, range]	0.27 ± 0.13 (0.05 – 0.7)
Phase I (0 ≤ rD50 < 0.25) [n per phase, %];	I: 34, 40%
Phase II (0.25 ≤ rD50 < 0.5) [n per phase, %];	II: 48, 56.4%
Phase III/IV (rD50 ≥ 0.5) [n per phase, %]	III: 3, 3.5%
D50 [months, mean ± SD, range]	36.3 ± 28.7 (8.07 – 175.55)
cFS [points, mean ± SD, range]	38 ± 6.4 (15.1 – 48.6)
cFL [points lost per month, mean ± SD, range]	0.89 ± 0.57 (0.11 – 2.85)
King's Stage [n per stage I-V]	I: 28
	II: 28
	III: 20
	IV: 9
	V: 0
MiToS Stage [n per stage 0-V]	0: 59
	I: 21
	II: 4
	III: 1
	IV/V: 0

*Abbreviations: ALSFRS-R* revised ALS functional rating scale (assessed within 100 days prior to or after MRI acquisition); *D50* estimated time in months for an individual to lose 50% of functionality; *PR* Progression Rate, calculated as (48 – current ALSFRS-R)/months since symptom onset; c*FS* calculated functional state, c*FL* calculated functional loss, *rD50* relative D50, each at the time of examination; *MiToS* Milano-Torino functional stage; *SD* standard deviation.

### 2.2 The D50 disease progression model

**Fig. 1:**
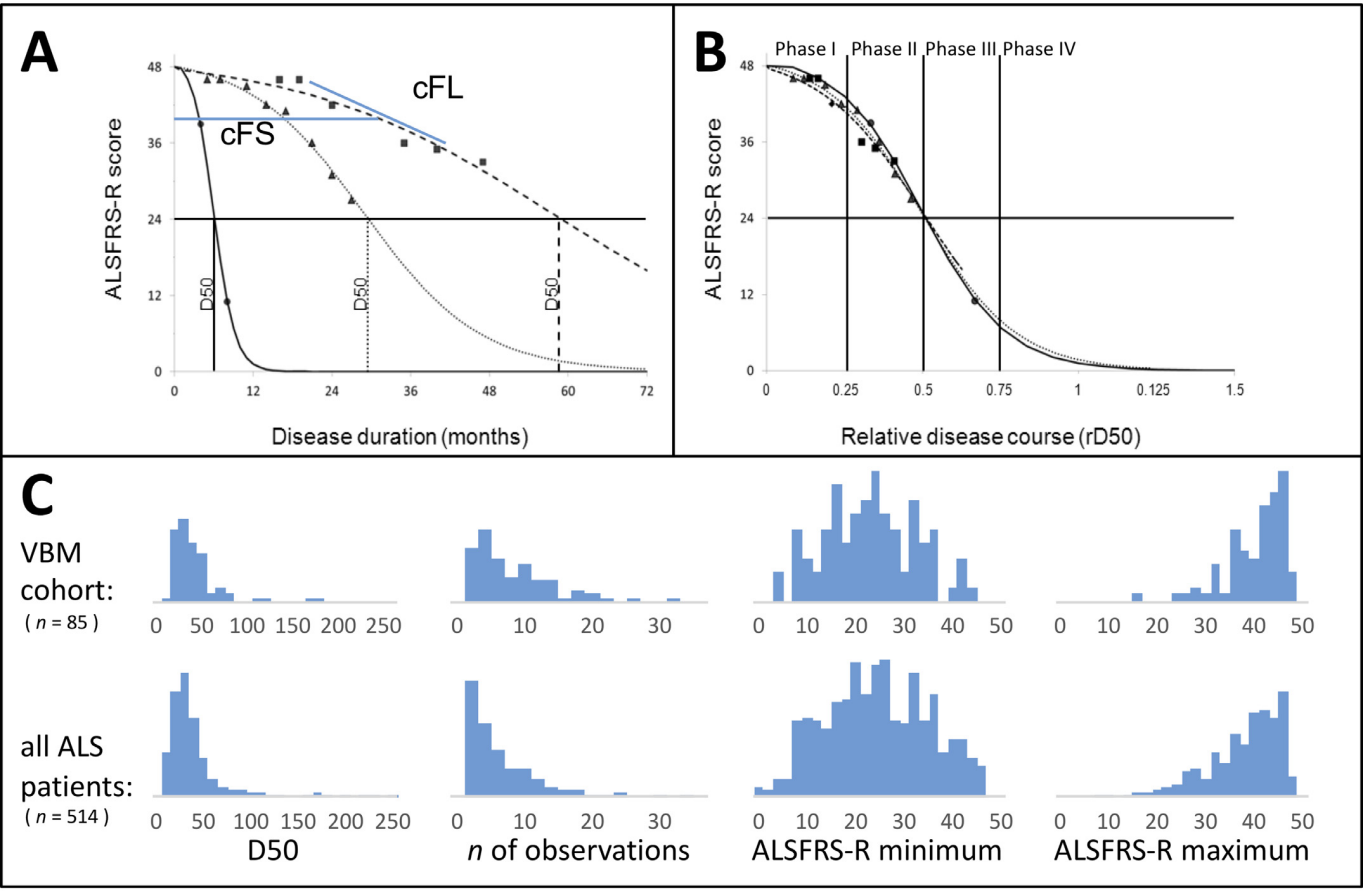
The D50 disease progression model: demonstration of principles and derived parameters. (A) Simulated disease curves of 3 ALS patients, with fast (circles), intermediate (triangles) and slow (squares) progression. Based on regularly obtained ALSFRS-R scores throughout the disease, the sigmoidal course is calculated. Resulting parameters: D50 = calculated time point when ALSFRS-R drops to 24, cFS = calculated functional state and cFL = calculated functional loss at the day of examination (for e.g. MRI acquisition). (B) Normalization with rD50, which describes individual disease course covered in reference to D50, allows for comparability between patients with vastly different disease aggressiveness and shows that patients proceed through similar Phases (I-IV) of functional decline. (C) Shows histograms of relevant variables for the current cohort of this study (top row) and all ALS patient data that is available in our center respectively. From left to right, the D50 values, number of obtained ALSFRS-R scores per patient, the minimum ALSFRS-R score and the maximum ALSFRS-R score obtained are noted; it illustrates, that the characteristics available for the VBM cohort well represent the regional ALS population. *Abbreviations: ALSFRS-R:* ALS Functional Rating Scale (Revised); *D50:* time until functionality drops to a half as estimated by the model; *cFS:* calculated functional state; *cFL:* calculated functional loss; *MRI*: Magnetic Resonance Imaging; *rD50:* relative D50.

Briefly, the D50 model describes the disease course of individual ALS patients as a sigmoidal state transition from full health to complete functional loss. The curve is calculated using iterative fitting of regularly collected ALSFRS-R scores that are available for a given patient. As a result, the D50 model provides measures of overall disease aggressiveness, local disease activity, and individual disease covered. Fig. 1 provides an overview of the D50 model and its derived parameters. Interested readers may also refer to Poesen et al. (2017) and Prell et al. (2019). D50 is defined as the time taken in months for a patient to lose 50% of his/her functionality (equivalent to an ALSFRS-R score of 24 from a maximum possible of 48) and thus provides a unified descriptive measure of a patient's overall disease aggressiveness. Normalizing an individual's D50 value to 0.5 yields the parameter relative D50 (rD50). rD50 is an open-ended reference point where 0 signifies disease onset and 0.5 indicates the time-point of halved functionality. By abstracting D50 from the disease trajectories, rD50 provides a continuous individualized unitl-ess scale of individual disease covered, independent of disease aggressiveness; this enables the quantitative comparison of accumulated disease in patients with vastly different progression types. Using rD50 in a simplified approach, patients can be categorized into at least 3 contiguous phases: an early semi-stable Phase I (0 ≤ rD50 < 0.25), an early progressive Phase II (0.25 ≤ rD50 < 0.5), and late progressive and late stable Phases III/IV (rD50 ≥ 0.5*,* see Fig. 1B).

The model also yields two mathematically derived descriptors of local disease activity; calculated functional state (cFS, in units of the ALSFRS-R) and calculated functional loss rate (cFL, local decay rate in points lost per month) (Fig. 1A). These can be calculated at any given point across the patient's disease course, for e.g. first visit, or time of MRI acquisition. These are particularly useful as they can provide an estimation of functional deterioration at time points where no ALSFRS-R score for the patient may be available and reduce noise inherent to the ALSFRS-R (see Fig. 1A).

### 2.3 MRI data acquisition and preprocessing

T1-weighted high resolution MRI images were obtained with a FLASH 3D sequence on a 1.5 Tesla Siemens Sonata scanner acquiring 192 sagittal slices (repetition time = 15 ms, echo time = 5 ms, Flip Angle = 30°) and using a standard 4-channel head coil. The original DICOM images were converted into the Nifti format using Dcm2Nii (MRIcroN). The CAT12- toolbox was used for preprocessing and VBM analyses (http://www.neuro.uni-jena.de/cat/), as it provides substantial improvements relative to the standard algorithms of the Statistical Parametric Mapping software (SPM) (for e.g. segmentation without tissue priors, integration of Dartel normalization). T1 data segmentation was performed and resulting probability maps for Gray Matter (GM) and White Matter (WM) were spatially normalized into MNI space using the default toolbox settings. Smoothing was applied with a standard Gaussian Kernel of 8 mm. Implicit masking was applied using an absolute threshold of 0.2.

### 2.4 Statistical analysis

All analyses were performed using Matlab (version R2009b). Between group-comparisons for age were performed using a two-sample *t*-test and revealed a significant difference between groups (ALS: 60.1 ± 11.5 years, controls: 54 ± 12.8 years, *p* = 0.003). However, no significant differences in gender distribution were noted (ALS: 36 female, 49 male; controls: 36 female, 26 male; assessed with Chi-square test: *p* = 0.266).

Between-group VBM comparisons for both GM and WM were performed using analyses of covariance (ANCOVA) model estimation and calculated using the Threshold-Free Cluster Enhancement (TFCE) approach, as implemented in the TFCE toolbox (version 119, http://dbm.neuro.uni-jena.de/tfce) (Smith and Nichols, 2009).

Between-group comparisons were first made on a case-control level i.e. between ALS patients and healthy individuals. Total intracranial volume, age and gender were included in the ANCOVA analysis as nuisance co-variates.

The ALS cohort was further sub-divided to perform additional sub-group comparisons, based on either **a)** onset-type (limb or bulbar), **b)** rD50-derived disease phase at time of MRI acquisition (Phase I vs. Phase II) or **c)** disease aggressiveness. For **c)** patients were classified as either having high or low overall disease aggressiveness based on their individual D50 values (D50 < 30 months = high aggressiveness). Onset-type, D50, rD50, age, gender and total intracranial volume were included as nuisance co-variates if not used as primary read-outs. As an example: sub-group comparisons between patients from either Phase I or II were accordingly corrected for onset-type, age, gender, total intracranial volume and D50 as possible confounders.

Multiple regression analyses were performed to assess possible associations between selected variables; from hereon, these will be referred to as “correlations” to maintain consistency and readability. Given that ALS sub-group comparisons revealed progressive involvement of both GM and WM segments between Phase I and Phase II (part **b**), disease state descriptors (rD50 and cFS) were used for voxel-wise regressions within WM and GM. Additional masking was applied using the significant clusters revealed from prior control vs. patient analyses for GM and WM segments, respectively*.* Similarly, regression analyses for D50 and cFL with WM clusters were also performed, given that D50-based sub-group analyses (part **c)** revealed differences only in WM density.

D50, rD50, cFS and cFL were natural log-transformed for all analyses mentioned above to achieve normality (Rajagopalan et al., 2013).

Statistical significance was set at either *p* < 0.001 after Family-Wise Error (FWE) correction for group-level comparisons, or *p* < 0.05 after FWE correction and additional Bonferroni adjustment of *p*-values to account for multiple comparisons for regression analyses.

## 3 Results

### 3.1 Widespread GM and WM changes are evident in ALS

**Fig. 2:**
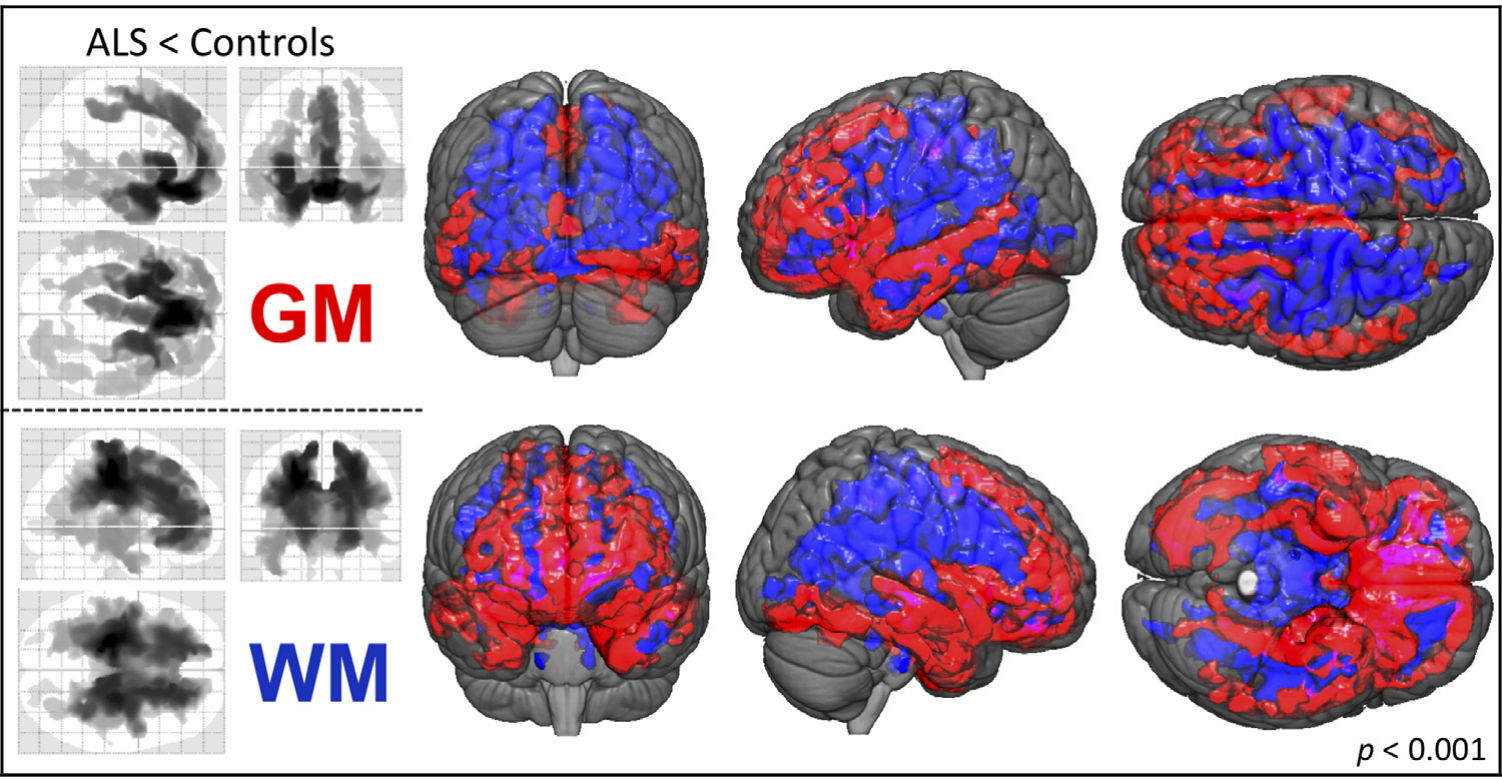
Between-group differences for patients and healthy controls. VBM between-group comparisons of patients (*n* = 85) and controls (*n* = 62) revealed GM density decreases (upper 3 glass-brain images, red-labeled in 3D-pictures) mainly outside the motor areas, i.e. within the bilateral frontal and temporal lobes. Observed WM density decreases in ALS patients (lower 3 glass-brain images, blue-labeled in 3D-pictures) encompassed major parts of the frontal structures and parietal projections underlying the motoric and somatosensoric cortices and extended throughout the brainstem. (TFCE; FWE corrected *p* < 0.001; nuisance co-variates: age, gender, total intracranial volume) *Abbreviations: GM:* Gray Matter*; TFCE:* Threshold-Free Cluster Enhancement*; FWE:* Family-Wise Error; *VBM*: Voxel-Based Morphometry; *WM:* White Matter.

At the case-control level, ALS patients showed significantly decreased GM density in the medial, inferior-frontal, and temporal lobes. Additional significant GM density decreases were also noted in the parietal, occipital and cerebellar and sub-cortical regions (FWE-corrected *p* < 0.001, Fig. 2, Supplementary Table 1A). No significant increases in GM density were observed between patients and controls.

WM density was decreased in patients primarily in the bilateral frontal and parietal regions, extending down to the brainstem. Additional decreases in WM density were also observed in the bilateral temporal lobes, but not within cerebellar projections (FWE-corrected *p* < 0.001, Fig. 2, Supplementary Table 2A). No significant WM density increases were noted between patients and controls. Sub-group comparisons showed that in contrast to patients with limb-onset, bulbar–onset patients presented with significantly decreased bifrontal and bitemporal GM and WM density (FWE-corrected *p* < 0.001, Supplementary Fig.1).

### 3.2 Structural changes across the phases of individual disease covered

**Fig. 3:**
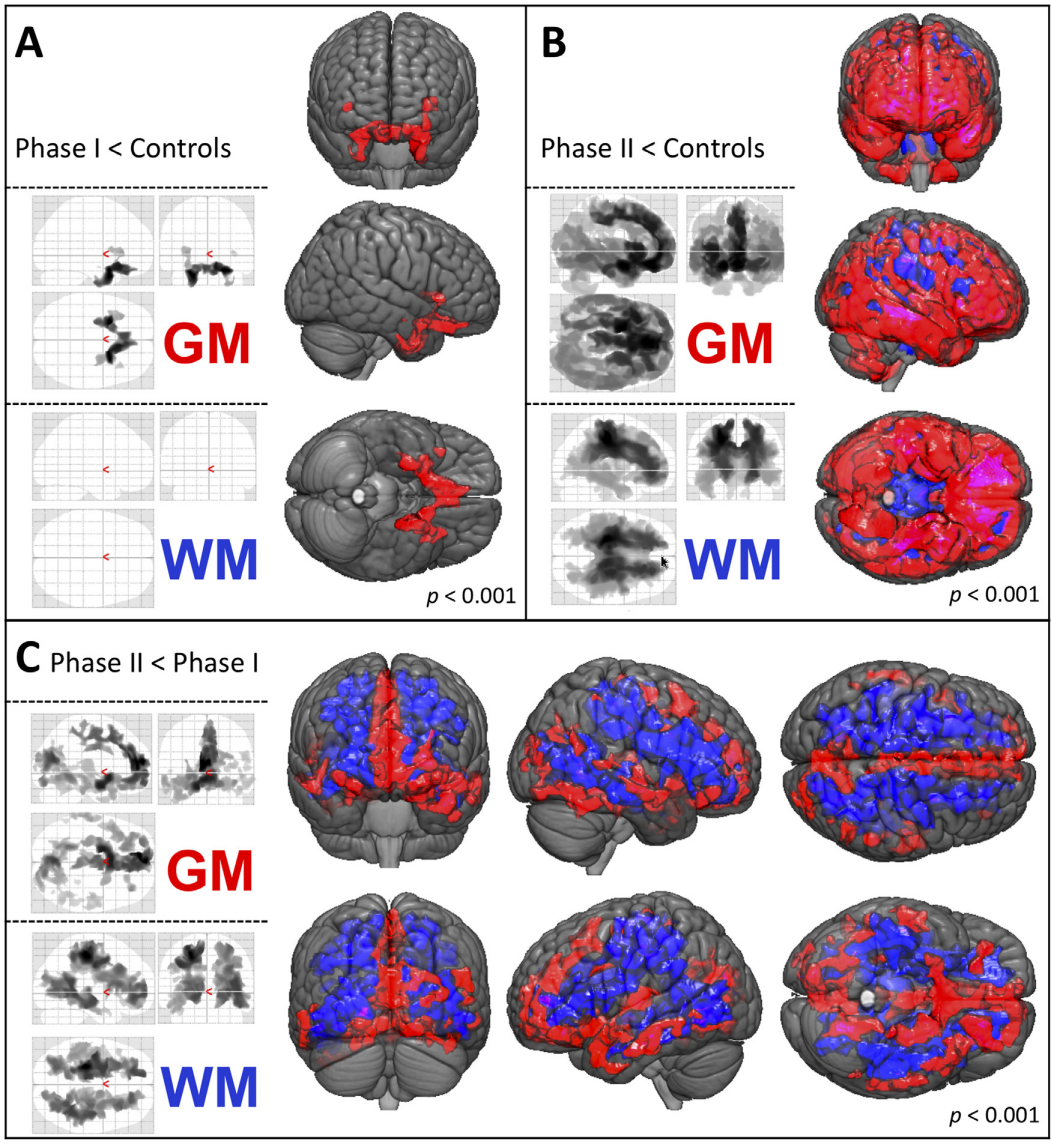
Comparisons across disease phases. VBM sub-group analyses of patients in disease Phases I (rD50 < 0.25, *n* = 34) and II (rD50 = 0.25–0.5, *n* = 48) at the time of MRI acquisition. (A) In Phase I GM density decreases were observed for inferior-frontal and temporal areas in contrast to healthy controls. (B) The Phase II patients showed an extended pattern of GM and WM atrophy as compared to healthy controls. (C) The direct comparison of both phases indicated that pathology spreads beyond the motor system, including frontal, temporal and occipital GM areas. The WM density decreases were even more extensive and emphasized in subparietal projections. (TFCE; FWE corrected *p* < 0.001; nuisance co-variates: age, gender, total intracranial volume, onset-type, D50) *Abbreviations: GM:* Gray Matter*; MRI:* Magnetic Resonance Imaging; *TFCE:* Threshold-Free Cluster Enhancement*; FWE:* Family-Wise Error; *VBM*: Voxel-Based Morphometry; *WM;* White Matter.

Decreases in GM density relative to controls were already evident in patients in Phase 1 (*n* = 34) (FWE-corrected *p* < 0.001), indicating that these structural changes manifest early in the relative disease course. These decreases were observed in the bilateral inferior-frontal and temporal areas; conversely, no significant changes in WM density were noted (Fig. 3A). Patients in Phase II (*n* = 48) also showed GM density decreases in the parietal, occipital and cerebellar regions relative to controls, as well as widespread WM density decreases (FWE-corrected *p* < 0.001, Fig. 3B).

Direct sub-group comparisons between patients in Phases I and II showed that the spread of structural changes mainly extended to the frontal, temporal and occipital GM areas, as well as within supratentorial WM projections (FWE-corrected *p* < 0.001, Fig. 3C, Supplementary Table 1B and 2B). There was no increased WM or GM density between ALS patients in Phases I and II relative to healthy controls or in patients in Phase II relative to those in Phase I.

Although regression analyses noted a negative correlation between rD50 and GM density in 3 small clusters (in the inferior-frontal gyrus, temporal pole and medial temporal cortex) in the left hemisphere, this failed to reach statistical significance after application of the Bonferroni post-hoc test. No significant associations between rD50 and WM density or cFS and GM/WM density were noted.

### 3.3 ALS-associated structural changes are influenced by disease aggressiveness

**Fig. 4:**
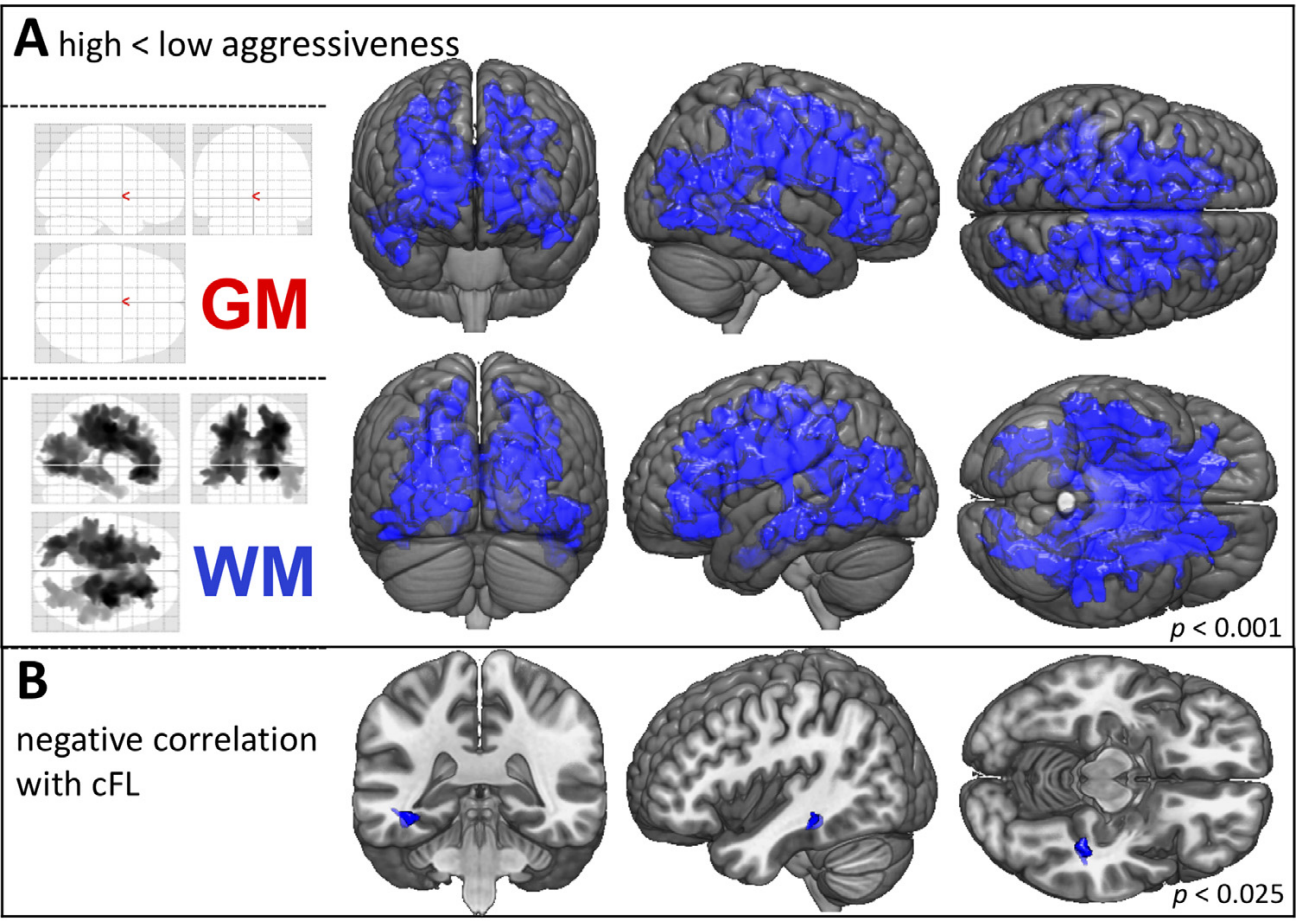
WM density decreases are associated with increased disease aggressiveness. A) VBM subgroup analyses of patients with high aggressive (*D50* < 30 months, *n* = 44) vs. low aggressive ALS (D50 >= 30 months, *n* = 41). High aggressiveness was associated with widespread WM volume-loss. The GM density was not influenced by disease aggressiveness. (TFCE; FWE corrected *p* < 0.001; nuisance co-variates: age, gender, total intracranial volume, onset-type, rD50). B) Voxel-wise regression revealed a negative correlation between WM density and cFL at the time of MRI acquisition, which was located in parts of the left inferior longitudinal and fronto-occipital fasciculus. (TFCE; FWE corrected *p* < 0.025; nuisance co-variate: total intracranial volume). *Abbreviations: GM:* Gray Matter*; cFL:* calculated functional loss; *TFCE:* Threshold-Free Cluster Enhancement*; FWE:* Family-Wise Error; *MRI:* Magnetic Resonance Imaging; *VBM*: Voxel-Based Morphometry; *WM:* White Matter.

Patients with high disease aggressiveness (*n* = 44) (D50 < 30 months) displayed a distinct pattern of supratentorial WM density decreases relative to those with low aggressiveness (*n* = 41) in direct sub-group comparisons (FWE-corrected *p* < 0.001). However, no significant differences in GM density were observed between these two groups (Fig. 4A, Supplementary Table 2C).

An inverse correlation between cFL and WM density alterations in left-hemispheric long association tracts connecting the frontal, parietal and occipital lobes, including parts of the superior longitudinal fasciculus, inferior longitudinal fasciculus and the fronto-occipital fasciculus, was observed in the regression analysis (FWE-corrected *p* < 0.025, Fig. 4B).

## 4 Discussion

To the best of our knowledge, the present study is the first to comprehensively evaluate neuroimaging correlates of ALS pathology within the context of disease aggressiveness (as defined and evaluated by the novel D50 model) in a large cohort of well-characterized patients.

Case-control comparisons between patients and healthy controls revealed distinct patterns of decreases in GM density. These were located primarily outside the classic motor areas. The most significant and widespread differences were noted in the medial and inferior parts of the frontal lobe, as well as within the temporal lobes. Additionally, parts of the parietal and occipital lobe, and sub-cortical regions were involved (Fig. 2, Supplementary Table 1A). Taken together, the results show augmented GM density decreases within the frontal and temporal lobes, which is in line with the results reported by prior cross-sectional studies and underscores the pathological overlap between ALS and frontotemporal dementia (Agosta et al., 2007; Christidi et al., 2018b; Rajagopalan and Pioro, 2015a; Trojsi et al., 2015). While some studies have used various neuropsychological indicators to demonstrate an association between frontal and/or temporal atrophy patterns and executive/behavioral symptoms (Bueno et al., 2018; Chang et al., 2005; Crespi et al., 2018; Mioshi et al., 2013), still others have reported that these frontotemporal alterations persist in the absence of measurable cognitive impairment (Bede et al., 2013a; Christidi et al., 2018a; Kim et al., 2017a). While patients in this cohort were clinically non-demented, no thorough neuropsychological testing was conducted. Therefore, we cannot entirely rule out that some patients may have had sub-clinical cognitive impairment, particularly since ALS patients often present with it (Abrahams et al., 2014; Goldstein and Abrahams, 2013; Montuschi et al., 2015; Ye et al., 2019). Neurocognitive deficits in ALS have been associated with structural GM changes in both temporal and occipital regions (Christidi et al., 2018a; Mezzapesa et al., 2007). Conversely, studies have also reported temporal and/or occipital GM changes in patients with normal neuropsychological profiles (Bede et al., 2013a, 2013b; Christidi et al., 2018a; Kassubek et al., 2005; Mezzapesa et al., 2013), suggesting that the effects of pathological changes in these regions extend beyond cognition. Correspondingly, Christidi et al. (2018b) described associations between GM density decreases in the occipital and temporal regions and transcranial magnetic stimulation parameters. They further postulated that this might reflect corticospinal neuronal involvement, which could by extension compromise fine-motor functions (i.e. visuomotor and visceromotor control) in ALS. We observed here that subcortical GM structures, including the bilateral amygdala and smaller portions of both thalami, were also altered in ALS patients, as has been previously demonstrated by case-control studies (Alruwaili et al., 2018; Bae et al., 2016; Bede et al., 2013b; Buhour et al., 2017; Chang et al., 2005; Christidi et al., 2018b; Kim et al., 2017a; Menke et al., 2018; Senda et al., 2017; Thivard et al., 2007). We also observed GM density decreases within both hippocampi (Abdulla et al., 2014; Bede et al., 2013a; Machts et al., 2015; Menke et al., 2018; Thivard et al., 2007; Westeneng et al., 2015) and both entorhinal cortices; these typically relay hippocampal input (Bueno et al., 2018). Additionally, we observed bilateral GM density decreases in the upper regions of the cerebellum, including cerebellar nuclei; cerebellar involvement has also been previously reported (Bae et al., 2016; Bueno et al., 2018; Buhour et al., 2017; Christidi et al., 2018a, 2018b; Kim et al., 2017a, 2017b; Thivard et al., 2007).

We observed pronounced and widespread WM density decreases in patients that were particularly accentuated within the frontal and temporal lobes (Fig. 2, Supplementary Table 2A). Former VBM studies have reported circumscribed clusters of decreases in WM density that were often located either next to the motor-cortex, in adjacent areas or within the corticospinal tract (De Marco et al., 2015; Kassubek et al., 2005; Kim et al., 2017a; Tsujimoto et al., 2011). A recent meta-analysis confirmed that the most significantly affected clusters were primarily within the supplementary motor areas, the precentral gyri, and the cerebellum (Chen et al., 2018a). However, some previous studies using classical VBM approaches reported no reductions in either GM (Acosta-Cabronero et al., 2018; Buhour et al., 2017) or WM density (Chang et al., 2005; Grosskreutz et al., 2006; Thivard et al., 2007) in patients with ALS. The reasons for these conflicting results might be partly explained by the heterogeneity in patients’ disease characteristics (Kim et al., 2017b), as well as the used methodology (Rajagopalan et al., 2013). The prominent WM pathology we observed in our study is likely a result of the specific T1-sequences used, as well as the TFCE approach (Rajagopalan and Pioro, 2015b).

Stratifying patients by onset-type revealed a distinct pattern of frontotemporal involvement in bulbar-onset patients (Supplementary Fig.1) which is in line with former studies (Chen et al., 2018b; Kim et al., 2017a). Studies have also reported that WM damage in bulbar-onset patients extends into the frontal and parietal regions and quite specifically follows the corticospinal tract (Ellis et al., 2001; Hartung et al., 2014). In summary, bulbar-onset patients show discrete and disparate structural changes that may be linked to rapid “neurodegenerative spread” and poorer clinical prognoses (Calvo et al., 2017; Marin et al., 2016). To account for this, onset-type was classified as a confounder in all other sub-group analyses.

### 4.1 Progressive GM and WM pathology occurs in disease Phase II

The application of the D50 model to this ALS cohort allowed the allocation of patients to contiguous albeit distinct disease phases (derived from rD50). We observed an evolution of structural changes from the early-stable Phase I to the early-progressive Phase II. Studies using six-monthly acquired MRIs noted longitudinal density changes in the precentral gyri, cingulate cortex and basal ganglia (Menke et al., 2014, 2018). However, Senda et al. (2011) reported decreases in volume, particularly in the frontal and temporal lobes. Using bigger time intervals, Agosta et al. (2009) reported a significant loss of structural integrity in the left premotor cortex and in the right basal ganglia at a 9-month follow-up, whilst Kwan et al. (2012) demonstrated cortical thinning and GM volume loss of the precentral gyri over an average interval of 1.26 years. These results align well with the structural changes observed across different rD50-derived disease phases here (Fig. 3), suggesting that they aren't merely a function of elapsed time but also reflect steadily declining functionality. To the best of our knowledge, application of the D50 model yields the strongest association between individual disease covered and VBM changes reported so far. Established staging systems for the disease course primarily rely on clinical milestones. The King's and MITOS staging systems (Roche et al., 2012;Chio et al., 2015) are the most frequently used, and can also be retrospectively calculated using available ALSFRS-R scores (Balendra et al., 2014; Fang et al., 2017). Prior criticisms of the MiToS system have stated that given its insensitivity to alterations early in the course of the disease, it may not be able to pick up on subtle neuroimaging correlates (Cardenas-Blanco et al., 2016; Ferraro et al., 2016). A cross-sectional analysis of patients using the King's system revealed GM changes in patients as early as stage 2A (close to diagnosis). Minor GM changes in the left inferior-frontal gyrus and right cuneus were observed in stage 2B patients (impairment of two regions) relative to healthy controls, while patients in stage 3 showed no significant GM changes (Trojsi et al., 2015).

Generally, ALS pathological spread is proposed to follow a corticofugal axonal alignment; four pathological stages have been proposed based on the spatial extent of abnormally phosphorylated TDP-43 inclusions within the brain (Brettschneider et al., 2013; Eisen et al., 2017). Interestingly, we observed no density alterations in central regions in patients in Phase I relative to controls, which may in part be because of the TFCE method we employed (t-contrasts revealed 2 small clusters in GM and adjacent WM here, for uncorrected *p* < 0.001). Existing reports of volumetric changes in the motor cortex while substantial, have mostly been inconsistent in their findings (Chio et al., 2014). In contrast, cortical thinning analyses have consistently demonstrated the involvement of primary motor areas (Agosta et al., 2012; Schuster et al., 2013; Walhout et al., 2015). Summarily speaking, the present study confirms that ALS-associated pathological changes originate cortically and are subsequently propagated along axonal projections.

In accordance with this, we also observed progressive involvement of sub-cortical GM structures; this was particularly pronounced in the left hemisphere for the amygdala, thalamus and hippocampus (Supplementary Table 1B). Hippocampal volume reductions in ALS patients have been reported (Abdulla et al., 2014; Bede et al., 2013a, 2013b). Moreover, Machts et al. (2015) described density and shape changes that were more pronounced in the presence of cognitive deficits. Westeneng et al. (2015) noted a progressive volumetric reduction in both hippocampi (left presubiculum and right CA2/3 and CA4/dentate gyrus) at a median follow-up interval of 5.5 months. Hippocampal volume changes, particularly those in the left hippocampus, correlated with performance on a verbal memory test (Abdulla et al., 2014; Machts et al., 2015).

Between-phase comparisons showed that WM changes were measurably evident in Phase II only. This is in agreement with a previous study that used voxel-based-intensometry (optimized approach for WM structures) and reported T1-related WM changes in a smaller ALS cohort. Here, increasing WM intensity alterations were observed between patients with high and low ALSFRS-R scores; these alterations also correlated with the current ALSFRS-R score in multiple levels of the corticospinal tract (Hartung et al., 2014). Overall, dividing patients into phases based on their individual functional loss profiles i.e. rD50-derived phases, revealed progressive cerebral alterations.

### 4.2 Extensive WM pathology is related to disease aggressiveness

As a next step, we checked for associations between VBM assessments of structural integrity and disease aggressiveness. Comparisons between patients with either low (D50 ≥ 30 months) or high (D50 < 30 months) overall aggressiveness revealed widespread supratentorial WM density decreases in the latter group (Fig. 4A). However, no differences were observed for GM density.

Disease progression in ALS has typically been described and quantified using the PR. However, the index presumes that progression in ALS is linear. It doesn't fully reflect the substantial inter- and intra-individual heterogeneity that patients present with over time; this effect is more pronounced in patients who progress rapidly. Given this, it is no surprise that previous reports of associations with PR haven't been as robust; Zhang et al. (2014) reported that a preselection of patients was needed to obtain an inverse correlation between GM density (in the right precentral gyri) and PR (only in patients with right limb-onset). Other studies noted no correlations between VBM-assessed alterations in either GM or WM and the PR (Grosskreutz et al., 2006; Rajagopalan et al., 2013; Rajagopalan and Pioro, 2015a; Zhu et al., 2015). Eventually, studies used an acute or “local” PR; for instance, Senda et al. (2017) defined 3 progression groups based on the extent of ALSFRS-R decline over a 6-month-follow-up interval. In doing so, they were able to demonstrate extensive involvement of the extra-motor cortex and basal ganglia in their rapid-progressive group.

Based on the results observed in the current study, VBM-based GM analyses may not be particularly sensitive to overall disease aggressiveness (D50) or calculated functional loss rate (cFL) at the time of MRI. Instead, the data reported here suggest that aggressiveness in ALS is primarily linked to WM pathology, which is in line with models that posit that pathology spreads via axonal projections throughout the brain (Brettschneider et al., 2013; Eisen et al., 2017). The WM changes observed in patients with highly aggressive disease may potentially underscore the increased vulnerability of these supratentorial neuronal tracts. No alterations were observed in the brainstem in these patients (although affected in the ALS cohort of this study, see Fig. 2), which is curious given that the brainstem is implicated in the early/initial pathological stages of the spreading model (pTDP-43 Stage 1). Furthermore, we noted significant correlations between WM density and cFL at the time of examination (Fig. 4B). These clusters were located in the long left-hemispheric association tracts, which have been proposed as mediators of pathological spreading in pTDP-43 stage 3 (Braak et al., 2013). The association between increased cFL and decreased WM integrity observed in this cross-sectional cohort highlights the potential neuroimaging biomarkers have to track clinical disease progression. Identifying patients with aggressive disease is critical for stratification for several health-care decisions, including follow-up intervals, therapeutic regimen, and assisted care, all of which have been shown to significantly influence morbidity and survival (Calvo et al., 2017; Paipa et al., 2019). Here, we postulate that a stronger association between the cFL and WM-tract changes may be more evident with methods that enable more detailed insight into microstructural changes of neuronal fiber tracts, such as for e.g. Diffusion Tensor Imaging (DTI) (Chio et al., 2014; Pradat and El Mendili, 2014; Zhang et al., 2018).

While the WM alterations described in our sub-group and multiple regression analyses are robust, they naturally require confirmation in larger, true longitudinal data sets. Large-scale multi-center studies would provide the necessary statistical power to confirm the current results (Filippi et al., 2015; Steinbach et al., 2018; Turner et al., 2011). One advantage of the D50 model lies in its easy applicability for any study or biomarker, because it only requires a set of properly ascertained ALSFRS-R scores per patient, and can thus also be utilized retrospectively.

## 5 Conclusions and limitations

The present study used the D50 progression model to better characterize a cross-sectional cohort and generate pseudo-longitudinal data. Our data suggest that while ALS is associated with substantial morphometric alterations in GM (as assessed by VBM), these do not fully reflect or associate with disease aggressiveness and are therefore unlikely candidates for clinical prognostication tools. However, alterations in WM structures seem to be strongly associated with increased disease aggressiveness. This is in keeping with the notion of ALS being a disease that despite having its onset foci in cortical areas, goes on to spread through axonal projections, and subsequently affects multiple domains of the central nervous system.

The present study is not without limitations. Most significantly, we were unable to conduct an extensive neuropsychological assessment of included participants, which would have undoubtedly shed light on the clinical implications of the substantial frontotemporal area alterations noted in the cohort. Given that this study only employed VBM to investigate structural changes in ALS, additional methods for e.g. Diffusion Tensor Imaging within WM regions would help to identify underlying tract damage. As with the majority of ALS studies, the recruited cohort was heterogeneous in terms of phenotype and disease duration; nevertheless, the D50 model minimizes the effect of the latter variable as it enables comparability between patients regardless of when they were recruited and assessed. The application of the D50 model to this cohort enabled detection of a strong association between ALS-related disease accumulation and in-vivo measures of T1-data derived structural alterations, which may otherwise have been obfuscated by disease heterogeneity. The model therefore lends itself and is easily applicable to clinical studies investigating neuroimaging or other correlates of ALS pathology and provides the opportunity to gain insight into disease-course related changes in otherwise cross-sectional cohorts. We recommend that further studies looking at, developing and validating neuroimaging or other biomarkers for ALS continue to work with ever larger, well-defined and multi-modal data sets.

## Funding

The present study was supported by the German Bundesministerium für Bildung und Forschung (BMBF) grant SOPHIA and ONWebDUALS to JG under the aegis of the EU Joint Programme Neurodegenerative Disease Research (JPND) and a BMBF grant PYRAMID to JG in the framework of the ERANET E-RARE program. Support was also received from the Motor Neurone Disease Association (MNDA) and Deutsche Gesellschaft für Muskelkranke (DGM). NG is supported by a doctoral scholarship (Landesgraduiertenstipendien) from the Graduate Academy of Friedrich Schiller University, Jena, Germany

## Declaration of Competing Interest

The authors have no conflicts of interest to declare.

## Appendix Supplementary materials

Image, application 1
