# Supplementary material for: Applying the D50 disease progression model to gray and white matter pathology in amyotrophic lateral sclerosis
Source: Neuroimage Clin. 2019 Nov 28;25:102094. doi: 10.1016/j.nicl.2019.102094 (PMC6940701; doi:10.1016/j.nicl.2019.102094)
Supplement: Supplementary file 1 [file mmc1.docx]

**Supplementary material**

Supplementary Table 1

| Anatomical labels | | l/r | region | percentage of this area activated | Voxel |
| --- | --- | --- | --- | --- | --- |
| **A) ALS < Controls** |  |  |  |  |  |
| frontal | Orbitofrontal cortex | l | Area Fo1 | 71.6 | 2661 |
|  |  |  | Area Fo2 | 80.4 | 2967 |
|  |  |  | Area Fo3 | 47 | 3918 |
|  |  | r | Area Fo1 | 73.9 | 2929 |
|  |  |  | Area Fo2 | 91.1 | 3020 |
|  |  |  | Area Fo3 | 51.1 | 4504 |
|  | Frontal pole | l | Area Fp1 | 47.9 | 6906 |
|  |  |  | Area Fp2 | 67 | 3877 |
|  |  | r | Area Fp1 | 29.1 | 3967 |
|  |  |  | Area Fp2 | 78.2 | 3822 |
|  | Precentral gyrus | r | Area 4a | 0.1 | 239 |
|  | Broca | l | Area 45 | 5.8 | 327 |
|  |  |  | Area 44 | 0.9 | 67 |
|  | Basal forebrain | l | BF (Ch 1-3) | 34.9 | 89 |
|  |  | r | BF (Ch 1-3) | 25.8 | 54 |
|  | Cingulum | l | Area s24 | 53.4 | 724 |
|  |  |  | Area 25 | 97.5 | 575 |
|  |  |  | Area s32 | 30.5 | 514 |
|  |  |  | Area 33 | 14.2 | 241 |
|  |  | r | Area s24 | 56.6 | 660 |
|  |  |  | Area 25 | 99.8 | 429 |
|  |  |  | Area s32 | 37.3 | 434 |
|  |  |  | Area 33 | 11.3 | 195 |
|  | Insula | l | Area Id1 | 25.1 | 236 |
|  |  | r | Area Id1 | 12.9 | 169 |
|  |  |  | Area Ig2 | 2.1 | 27 |
|  |  |  |  |  |  |
| temporal/ subcortical | Auditory cortex | l | Area TE 3 | 1.3 | 89 |
|  |  | r | Area TE 1 | 0.9 | 11 |
|  |  |  | Area TE 1.2 | 4.7 | 39 |
|  |  |  | Area TE 3 | 6.5 | 541 |
|  | Entorhinal Cortex | l | Entorhinal Cortex | 88.5 | 2013 |
|  |  | r | Entorhinal Cortex | 52.5 | 1346 |
|  | Hippocampus | l | CA1 (Hippocampus) | 38.8 | 675 |
|  |  |  | CA2 (Hippocampus) | 11.7 | 51 |
|  |  |  | CA3 (Hippocampus) | 25.5 | 83 |
|  |  |  | HATA Region | 66.1 | 125 |
|  |  |  | Subiculum | 50.7 | 1539 |
|  |  | r | CA1 (Hippocampus) | 37.3 | 854 |
|  |  |  | CA2 (Hippocampus) | 12.7 | 62 |
|  |  |  | CA3 (Hippocampus) | 23.8 | 3 |
|  |  |  | DG (Hippocampus) | 16.5 | 172 |
|  |  |  | Subiculum | 31.2 | 944 |
|  | Amygdala | l | Amygdala (SF) | 76.7 | 224 |
|  |  |  | Amygdala (CM) | 28.8 | 100 |
|  |  |  | Amygdala (LB) | 95.1 | 1843 |
|  |  | r | Amygdala (SF) | 37.2 | 144 |
|  |  |  | Amygdala (CM) | 21.2 | 47 |
|  |  |  | Amygdala (LB) | 95.3 | 1619 |
|  | Thalamus | l | Thal: Prefrontal | 4.7 | 238 |
|  |  | r | Thal: Temporal | 8.8 | 384 |
|  |  |  |  |  |  |
| occipital | Striate cortex | l | Area hOc1 [V1] | 0.7 | 116 |
|  | Extrastriate cortex | l | Area hOc2 [V2] | 1.4 | 106 |
|  |  |  | Area hOc4v [V4(v)] | 4 | 233 |
|  |  | r | Area hOc3v [V3v] | 12 | 820 |
|  |  |  | Area hOc4la | 7.5 | 534 |
|  |  |  | Area hOc4v [V4(v)] | 31.9 | 1589 |
|  | Fusiform gyrus | l | Area FG1 | 4.3 | 88 |
|  |  |  | Area FG2 | 10.2 | 413 |
|  |  |  | Area FG3 | 25.5 | 1203 |
|  |  |  | Area FG4 | 4.1 | 270 |
|  |  | r | Area FG1 | 20.3 | 404 |
|  |  |  | Area FG2 | 34.9 | 905 |
|  |  |  | Area FG3 | 18.4 | 720 |
|  |  |  | Area FG4 | 43.3 | 2272 |
|  |  |  |  |  |  |
| parietal | Superior parietal cotex | l | Area 5Ci (SPL) | 22.7 | 215 |
|  |  |  | Area 5M (SPL) | 7.4 | 285 |
|  |  |  |  |  |  |
| cerebellum | Cortex | l | Lobule VI (Hem) | 12.5 | 1886 |
|  |  |  | Lobule VIIa crusI (Hem) | 0.1 | 30 |
|  |  | r | Lobule V (Hem) | 0.4 | 25 |
|  |  |  | Lobule VI (Hem) | 26.3 | 3795 |
|  |  |  | Lobule VIIa crusI (Hem) | 5.3 | 1371 |
|  | Nuclei | l | Interposed Nucleus | 24.1 | 83 |
|  |  |  | Ventral Dentate | 18.9 | 75 |
|  |  | r | Interposed Nucleus | 11.6 | 34 |
|  |  |  |  |  |  |
|  |  |  |  |  |  |
| Anatomical labels | | l/r | region | percentage of this area activated | Voxel |
| **B) Phase II < Phase I** |  |  |  |  |  |
| frontal | Orbitofrontal cortex | l | Area Fo1 | 14.4 | 536 |
|  |  |  | Area Fo2 | 24.7 | 910 |
|  |  |  | Area Fo3 | 31.5 | 2626 |
|  |  | r | Area Fo1 | 20.4 | 807 |
|  |  |  | Area Fo2 | 26.8 | 888 |
|  |  |  | Area Fo3 | 11.2 | 992 |
|  | Frontal pole | l | Area Fp1 | 31.7 | 4569 |
|  |  |  | Area Fp2 | 71.1 | 4115 |
|  |  | r | Area Fp1 | 5.9 | 810 |
|  |  |  | Area Fp2 | 65.4 | 3200 |
|  | Precentral gyrus | l | Area 4a | 0.7 | 54 |
|  |  | r | Area 4a | 5.1 | 445 |
|  | Basal forebrain | l | BF (Ch 4) | 4.2 | 17 |
|  |  | r | BF (Ch 1-3) | 33 | 69 |
|  | Cingulum | l | Area s24 | 53.4 | 724 |
|  |  |  | Area 25 | 97.6 | 576 |
|  |  |  | Area s32 | 29.8 | 501 |
|  |  |  | Area 33 | 11.8 | 201 |
|  |  | r | Area s24 | 48.2 | 562 |
|  |  |  | Area 25 | 100 | 430 |
|  |  |  | Area s32 | 10.9 | 127 |
|  |  |  | Area 33 | 27 | 467 |
|  | Insula | r | Area Id1 | 8.1 | 106 |
|  |  |  | Area Ig2 | 2 | 25 |
|  |  |  |  |  |  |
| temporal/subcortical | Auditory cortex | r | Area TE 1 | 0.2 | 3 |
|  |  |  | Area TE 3 | 0.2 | 16 |
|  | Entorhinal cortex | l | Entorhinal Cortex | 18.6 | 424 |
|  | Hippocampus | l | CA1 (Hippocampus) | 23 | 400 |
|  |  |  | CA2 (Hippocampus) | 2.8 | 12 |
|  |  |  | CA3 (Hippocampus) | 14.2 | 46 |
|  |  |  | DG (Hippocampus) | 15.1 | 201 |
|  |  |  | Subiculum | 26.9 | 815 |
|  | Amygdala | l | Amygdala (LB) | 79.9 | 1549 |
|  |  |  | Amygdala (SF) | 63.4 | 185 |
|  |  |  | Amygdala (CM) | 33.1 | 115 |
|  |  | r | Amygdala (LB) | 22.2 | 377 |
|  | Thalamus | l | Thal: Prefrontal | 30.2 | 1527 |
|  |  |  | Thal: Temporal | 14.6 | 623 |
|  |  | r | Thal: Prefrontal | 3.6 | 161 |
|  |  |  | Thal: Temporal | 3.1 | 136 |
|  |  |  |  |  |  |
| occipital | Striate cortex | l | Area hOc1 [V1] | 14.4 | 2326 |
|  |  | r | Area hOc1 [V1] | 21.6 | 3571 |
|  | Extrastriate cortex | l | Area hOc2 [V2] | 14.5 | 1090 |
|  |  |  | Area hOc3v [V3v] | 0.3 | 20 |
|  |  |  | Area hOc3d [V3d] | 5.1 | 407 |
|  |  |  | Area hOc4v [V4(v)] | 2.6 | 149 |
|  |  | r | Area hOc2 [V2] | 21.7 | 1764 |
|  |  |  | Area hOc3v [V3v] | 8 | 547 |
|  |  |  | Area hOc3d [V3d] | 7.7 | 339 |
|  |  |  | Area hOc4v [V4(v)] | 8.5 | 423 |
|  |  |  | Area hOc4d [V3A] | 7.4 | 248 |
|  |  |  | Area hOc4la | 1.9 | 135 |
|  |  |  | Area hOc4lp | 14.9 | 663 |
|  | Fusiform gyrus | l | Area FG1 | 11.2 | 229 |
|  |  |  | Area FG3 | 22.5 | 1018 |
|  |  | r | Area FG1 | 19.4 | 386 |
|  |  |  | Area FG2 | 0.5 | 13 |
|  |  |  | Area FG3 | 8.8 | 344 |
|  |  |  | Area FG4 | 17.3 | 911 |
|  |  |  |  |  |  |
| parietal | Superior parietal cortex | l | Area 5M (SPL) | 0.5 | 21 |
|  |  |  | Area 7M (SPL) | 1.6 | 20 |
|  |  | r | Area 5Ci (SPL) | 23.2 | 368 |
|  |  |  | Area 5M (SPL) | 4 | 92 |
|  | Inferior parietal cortex | r | Area PGa (IPL) | 8.6 | 510 |
|  |  |  | Area PGp (IPL) | 2.2 | 175 |
|  |  |  |  |  |  |
| cerebellum | Cortex | l | Lobule I IV (Hem) | 1.6 | 63 |
|  |  |  | Lobule V (Hem) | 14.4 | 837 |
|  |  |  | Lobule VI (Hem) | 11 | 1659 |
|  |  | r | Lobule I IV (Hem) | 0.5 | 21 |
|  |  |  | Lobule V (Hem) | 0.7 | 46 |
|  |  |  | Lobule VI (Hem) | 14.2 | 2055 |
|  |  |  | Lobule VIIa crusI (Hem) | 3.5 | 915 |
| **C) high < low aggressiveness** *No surviving suprathreshold clusters* | | | | | |

Summary of relevant GM VBM group differences revealed between A) ALS patients and healthy controls, B) patients in disease Phases I (rD50 < 0.25, *n* = 34) and Phase II (rD50 = 0.25-0.5, *n* = 48), C) patients with high aggressive (D50 < 30 months, *n* = 44) vs. low aggressive ALS (D50 >= 30 months, *n* = 41). Regions have been identified with the Anatomy-toolbox (version 2.2b) in SPM 12, the region-labels in column 4 refer to the original output of the toolbox ([https://www.fz-juelich.de/inm/inm1/DE/Forschung/docs/SPMAnatomyToolbox/ SPMAnatomyToolbox_node.html](https://www.fz-juelich.de/inm/inm1/DE/Forschung/docs/SPMAnatomyToolbox/%20SPMAnatomyToolbox_node.html)). *Abbreviations:* *l*: left hemisphere; *r:* right hemisphere.

Supplementary Table 2

| Anatomical labels |  | | l/r | percentage of whole sign. cluster volume assigned to this region |
| --- | --- | --- | --- | --- |
| **A) ALS < Controls** | |  |  |  |
| Corpus callosum | | Genu of corpus callosum |  | 2.56 |
|  | | Body of corpus callosum |  | 4.09 |
|  | | Splenium of corpus callosum |  | 1.86 |
|  | | Tapetum | r | 0.11 |
|  | | Tapetum | l | 0.17 |
|  | |  |  |  |
| Fornix | | Fornix (column and body of fornix) |  | <0.01 |
|  | | Fornix (cres) / Stria terminalis (can not be resolved with current resolution) | r | 0.21 |
|  | | Fornix (cres) / Stria terminalis (can not be resolved with current resolution) | l | 0.25 |
|  | |  |  |  |
| Corticospinal tract | | Corticospinal tract | r | 0.02 |
|  | | Corticospinal tract | l | 0.09 |
|  | |  |  |  |
| Internal capsule | | Anterior limb of internal capsule | r | 0.15 |
|  | | Anterior limb of internal capsule | l | 0.34 |
|  | | Posterior limb of internal capsule | r | 0.88 |
|  | | Posterior limb of internal capsule | l | 0.79 |
|  | | Retrolenticular part of internal capsule | r | 0.52 |
|  | | Retrolenticular part of internal capsule | l | 0.44 |
|  | |  |  |  |
| External capsule | | External capsule | r | 0.76 |
|  | | External capsule | l | 0.81 |
|  | |  |  |  |
| Cingulum | | Cingulum (cingulate gyrus) | r | 0.23 |
|  | | Cingulum (cingulate gyrus) | l | 0.63 |
|  | | Cingulum (hippocampus) | r | <0.01 |
|  | |  |  |  |
| Corona radiata | | Anterior corona radiata | r | 2.07 |
|  | | Anterior corona radiata | l | 2.07 |
|  | | Superior corona radiata | r | 2.25 |
|  | | Superior corona radiata | l | 2.26 |
|  | | Posterior corona radiata | r | 0.98 |
|  | | Posterior corona radiata | l | 1.03 |
|  | |  |  |  |
| thalamic radiation | | Posterior thalamic radiation  (include optic radiation) | r | 0.59 |
|  | | Posterior thalamic radiation  (include optic radiation) | l | 0.97 |
|  | |  |  |  |
| Association tracts | | Sagittal stratum (include inferior longitidinal fasciculus and inferior fronto-occipital fasciculus) | r | 0.50 |
|  | | Sagittal stratum (include inferior longitidinal fasciculus and inferior fronto-occipital fasciculus) | l | 0.66 |
|  | | Superior longitudinal fasciculus | r | 1.72 |
|  | | Superior longitudinal fasciculus | l | 1.67 |
|  | | Superior fronto-occipital fasciculus | r | 0.14 |
|  | | Superior fronto-occipital fasciculus | l | 0.15 |
|  | | Uncinate fasciculus | r | <0.01 |
|  | | Uncinate fasciculus | l | <0.01 |
|  | |  |  |  |
| Brainstem | | Medial lemniscus | l | 0.01 |
|  | | Cerebral peduncle | r | 0.65 |
|  | | Cerebral peduncle | l | 0.59 |
|  | |  |  |  |
| Cerebellum | | Middle cerebellar peduncle |  | 0.17 |
|  | | Pontine crossing tract (a part of MCP) |  | <0.01 |
|  | | Superior cerebellar peduncle | r | 0.02 |
|  | | Superior cerebellar peduncle | l | 0.09 |
|  | |  |  |  |
| Unclassified | |  |  | 66.47 |
| Anatomical labels |  | | l/r | percentage of whole sign. cluster volume assigned to this region |
| **B) Phase II < Phase I** | |  |  |  |
| Corpus callosum | | Genu of corpus callosum |  | 0.73 |
|  | | Body of corpus callosum |  | 1.09 |
|  | | Splenium of corpus callosum |  | 3.43 |
|  | | Tapetum | r | 0.19 |
|  | | Tapetum | l | <0.01 |
|  | |  |  |  |
| Fornix | | Fornix (cres) / Stria terminalis | r | 0.28 |
|  | | Fornix (cres) / Stria terminalis | l | 0.05 |
|  | |  |  |  |
| Internal capsule | | Anterior limb of internal capsule | r | 0.74 |
|  | | Anterior limb of internal capsule | l | 1.23 |
|  | | Posterior limb of internal capsule | r | 0.13 |
|  | | Posterior limb of internal capsule | l | 0.27 |
|  | | Retrolenticular part of internal capsule | r | 0.32 |
|  | | Retrolenticular part of internal capsule | l | 0.1 |
|  | |  |  |  |
| External capsule | | External capsule | r | 0.99 |
|  | | External capsule | l | 0.55 |
| Cingulum | | Cingulum (cingulate gyrus) | r | 0.37 |
|  | | Cingulum (cingulate gyrus) | l | 0.43 |
|  | | Cingulum (hippocampus) | r | 0.06 |
|  | | Cingulum (hippocampus) | l | 0.05 |
|  | |  |  |  |
| Corona radiata | | Anterior corona radiata | r | 3.17 |
|  | | Anterior corona radiata | l | 2.38 |
|  | | Superior corona radiata | r | 2.2 |
|  | | Superior corona radiata | l | 2.84 |
|  | | Posterior corona radiata | r | 0.13 |
|  | | Posterior corona radiata | l | 0.75 |
|  | |  |  |  |
| Thalamic radiation | | Posterior thalamic radiation (include optic radiation) | r | 2.38 |
|  | | Posterior thalamic radiation (include optic radiation) | l | 1.33 |
|  | |  |  |  |
| Association tracts | | Sagittal stratum (include inferior longitidinal fasciculus and inferior fronto-occipital fasciculus) | r | 1.39 |
|  | | Sagittal stratum (include inferior longitidinal fasciculus and inferior fronto-occipital fasciculus) | l | 1.03 |
|  | | Superior longitudinal fasciculus | r | 1.25 |
|  | | Superior longitudinal fasciculus | l | 0.92 |
|  | | Superior fronto-occipital fasciculus | r | 0.17 |
|  | | Superior fronto-occipital fasciculus | l | 0.09 |
|  | | Uncinate fasciculus | l | <0.01 |
|  | |  |  |  |
| Brainstem | | Cerebral peduncle | r | 0.01 |
|  | |  |  |  |
| Unclassified | |  |  | 68.94 |
| Anatomical labels |  | | l/r | percentage of whole sign. cluster volume assigned to this region |
| **C) high < low aggressiveness** | | |  |  |
| Corpus callosum | | Genu of corpus callosum |  | 2.82 |
|  | | Body of corpus callosum |  | 5.43 |
|  | | Splenium of corpus callosum |  | 4.30 |
|  | | Tapetum | r | 0.09 |
|  | | Tapetum | l | 0.2 |
|  | |  |  |  |
| Fornix | | Fornix (cres) / Stria terminalis | r | 0.19 |
|  | | Fornix (cres) / Stria terminalis | l | 0.28 |
|  | |  |  |  |
| Internal capsule | | Anterior limb of internal capsule | r | 0.15 |
|  | | Anterior limb of internal capsule | l | 0.33 |
|  | | Posterior limb of internal capsule | r | <0.01 |
|  | | Posterior limb of internal capsule | l | 0.27 |
|  | | Retrolenticular part of internal capsule | r | 0.38 |
|  | | Retrolenticular part of internal capsule | l | 0.17 |
|  | |  |  |  |
| External capsule | | External capsule | r | 0.71 |
|  | | External capsule | l | 0.47 |
|  | |  |  |  |
| Cingulum | | Cingulum (cingulate gyrus) | r | 0.25 |
|  | | Cingulum (cingulate gyrus) | l | 0.76 |
|  | | Cingulum (hippocampus) | r | 0.01 |
|  | | Cingulum (hippocampus) | l | 0.04 |
|  | |  |  |  |
| Corona radiata | | Anterior corona radiata | r | 2.72 |
|  | | Anterior corona radiata | l | 2.2 |
|  | | Superior corona radiata | r | 2.92 |
|  | | Superior corona radiata | l | 3.19 |
|  | | Posterior corona radiata | r | 0.84 |
|  | | Posterior corona radiata | l | 1.24 |
|  | |  |  |  |
| Thalamic radiation | | Posterior thalamic radiation (include optic radiation) | r | 0.68 |
|  | | Posterior thalamic radiation (include optic radiation) | l | 1.21 |
|  | |  |  |  |
| Association tracts | | Sagittal stratum (include inferior longitidinal fasciculus and inferior fronto-occipital fasciculus) | r | 0.79 |
|  | | Sagittal stratum (include inferior longitidinal fasciculus and inferior fronto-occipital fasciculus) | l | 0.84 |
|  | | Superior longitudinal fasciculus | r | 1.57 |
|  | | Superior longitudinal fasciculus | l | 1.58 |
|  | | Superior fronto-occipital fasciculus | r | 0.16 |
|  | | Superior fronto-occipital fasciculus | l | 0.21 |
|  | |  |  |  |
| Unclassified | |  |  | 63.01 |

Summary of relevant WM VBM group differences revealed between A) ALS patients and healthy controls; B) ALS patients in disease Phases I (rD50 < 0.25, *n* = 34) and Phase II (rD50 = 0.25-0.5, *n* = 48); C) ALS patients with high overall disease aggressiveness (D50 < 30 months, *n* = 44) vs. low aggressiveness (D50 >= 30 months, *n* = 41). The WM regions have been identified based on the ICBM-DTI-81 white-matter labels atlas in the FMRIB Software Library (FSL, release 4.1, <https://fsl.fmrib.ox.ac.uk/fsl>). *Abbreviations:* *l*: left hemisphere; *r:* right hemisphere.


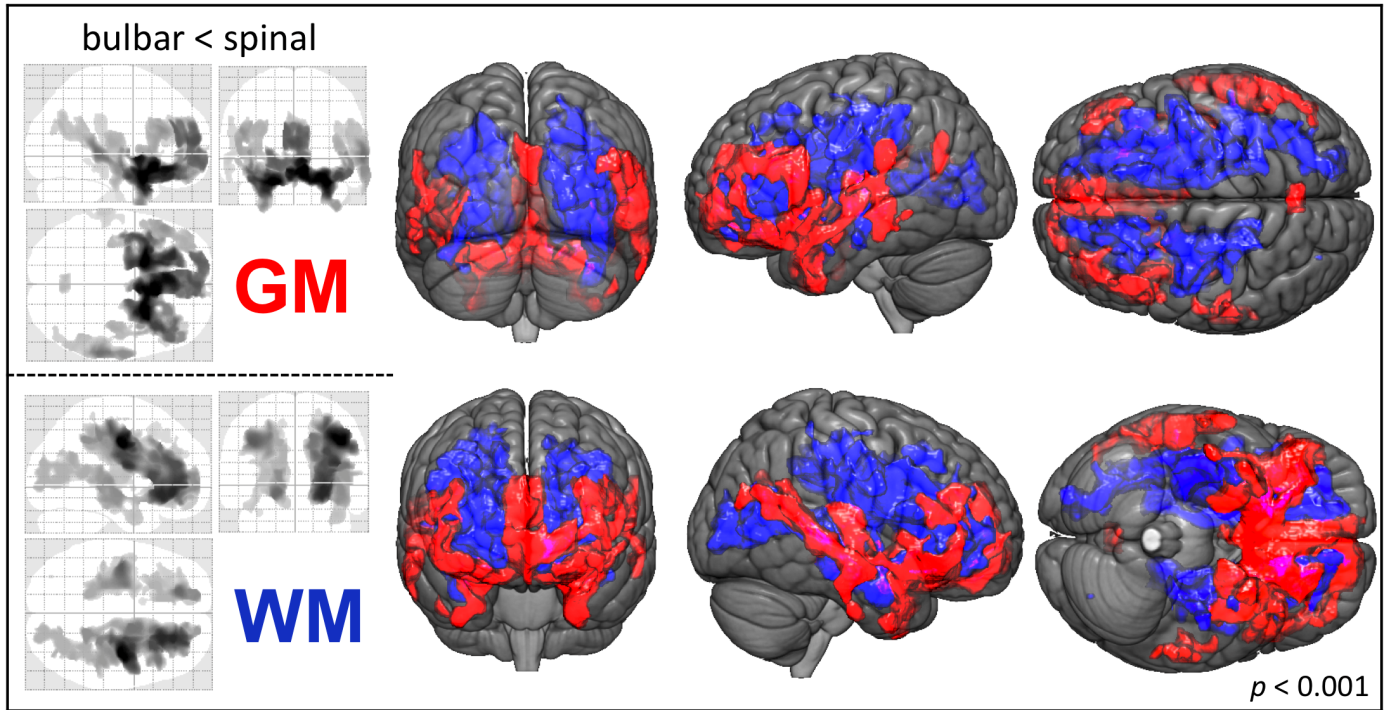


**Supplementary Figure 1**

**Onset-type comparisons**

VBM sub-group analyses of patients with either bulbar (*n* = 24) or spinal onset (*n* = 61). Bulbar–onset patients showed decreased bifrontal and bitemporal GM and WM density (TFCE; FWE corrected *p* < 0.001; nuisance co-variates: age, gender, total intracranial volume, rD50, D50), which is in line with former studies (Chen et al., 2018b; Ellis et al., 2001; Hartung et al., 2014; Kim et al., 2017a). Consequently, onset-type was included as a possible confounder in subsequent sub-group analyses.

*Abbreviations:* *GM:* Grey Matter*; TFCE:* Threshold-Free Cluster Enhancement*; FWE:* Family-Wise Error; *VBM*: Voxel-Based Morphometry; *WM:* White Matter.
